# Impaired cognitive modification for estimating time duration in Parkinson’s disease

**DOI:** 10.1371/journal.pone.0208956

**Published:** 2018-12-13

**Authors:** Motoyasu Honma, Yuri Masaoka, Shinichi Koyama, Takeshi Kuroda, Akinori Futamura, Azusa Shiromaru, Yasuo Terao, Kenjiro Ono, Mitsuru Kawamura

**Affiliations:** 1 Showa University School of Medicine, Department of Neurology, Shinagawa-ku, Tokyo, Japan; 2 Kyorin University School of Medicine, Department of Physiology, Mitaka-shi, Tokyo, Japan; 3 Showa University School of Medicine, Department of Physiology, Shinagawa-ku, Tokyo, Japan; 4 University of Tsukuba, School of Art and Design, Tsukuba, Ibaraki, Japan; Nathan S Kline Institute, UNITED STATES

## Abstract

Parkinson's disease (PD) is associated with various cognitive impairments. However, the nature of cognitive modification in patients with PD remains to be elucidated. In the present study, we examined whether patients with PD could correct and maintain subjective time duration and line length estimation. After training sessions, in which participants repeatedly memorized either a duration or a length, we compared a learning performance in 20 PD patients with 20 healthy controls. In the case of duration in the PD patients, the learned durations immediately returned to baseline of pre-training within a few minutes. However, the patients’ ability to learn length estimation remained unimpaired. In contrast, healthy controls were able to retain the learned duration and length estimations. Time compression in PD's internal clock may become entrained to their altered duration estimation even after learning of accurate time duration. These deficits may be associated with disrupting cognitive modification in PD.

## Introduction

The ability to accurately recognize time duration is fundamental to everyday life, and self-setting of time duration without cues is affected by various factors [1, 2]. Some neurological diseases, particularly Parkinson’s disease (PD), involve disordered temporal processing [3, 4]. Patients with PD have decreased levels of dopamine (DA), as well as various impairments to cognitive function [5–7]. In particular, they tend to underestimate time duration [8]. Administration of DA agonists causes a shift toward normal perception of time duration [9], indicating that DA levels are associated with time estimation, and therefore that the basal ganglia are involved in temporal processing [10, 11]. Moreover, striatal activity plays a major role in the acquisition and early maintenance stages of instrumental learning [12]. Patients with PD develop cognitive learning disabilities, including difficulties with decision-making, attention, and visual discrimination [13–16]. DA is a substrate for synaptic plasticity and memory formation [17], and it may be that patients with basal ganglia disorders cannot adjust their behavior to situational demands.

The learning mechanisms underlying estimation of time duration and length have not yet been examined, even in healthy individuals. Nonetheless, it follows that, if striatum deficits affected learning across cognitive tasks, patients with PD would experience dysfunctional estimation of both time duration and length. In contrast, if learning was only dysfunctional in one dimension, cognitive modification would specifically be lost in that dimension, rather than more generally. Because time duration estimation shows severe disruption [9, 18] while length estimation is normal in patients with PD [19], it may be that cognitive function is lost within that specific dimension. Therefore, the time durations produced by patients would immediately return to their baseline values, even after the patients had learned the correct time duration, while lengths produced by patients would remain accurate.

In the present study, we conducted behavioral experiments to test a learning ability in both time duration and line length estimations (“Estimation training task”) by comparison of PD patients with healthy controls (HCs). To examine memory ability, we confirmed that all participants could reproduce the line length and time duration after 5 minutes (“Delayed reproduction task”).

## Methods

### Standard protocol approvals, registration, and patient consents

This study was approved by the Ethics Committees of Showa University Hospital (clinical trial identifier number: 4192) and was conducted according to the Principles of the Declaration of Helsinki. All participants provided written informed consent.

### Participants

Sample size was determined by effect size in previous studies related to cognition and leaning [8–10,18,19]. In case-controlled study design, Clinical neurologists recruited 43 patients with PD who met the diagnostic criteria of the Parkinson’s Disease Society Brain Bank [20]. Among them, 20 were selected to participate in this study. Participants were included if they had no signs of dementia, as determined by two cognitive assessment batteries: the Mini-Mental Status Examination (MMSE; score > 25), which tests memory, attention, and language abilities [21]; and the Montreal Cognitive Assessment (MoCA; score > 25), which tests memory, visuospatial, executive, attention, concentration, and language abilities [22]. For both the MMSSE and MoCA, we used a cut-off score of “> 25”. We excluded any patients at the lower limit of either the MMSE or MoCA. We also recruited 26 elderly HCs with no history of neurological disease and no signs of dementia. Among them, we excluded six who overestimated time duration (by judging objective 22 s duration as more than 23 s subjectively). The learning effect may take different meanings, in that an overproduced person learned a standard duration of 22 s (toward short duration) from in that an underproduced person learned the duration (toward long duration). Along with the reason that overproduced persons were a small group, we excluded 6 HCs from the current analysis, to clear the effect.

There was no significant difference in age between the HC and PD groups, and all participants were right-handed (Table 1). Patients with PD and HCs showed no brain abnormalities on magnetic resonance imaging (MRI) with fluid-attenuated inversion recovery and diffusion-weighted imaging. PD severity was measured with the Unified Parkinson’s Disease Rating Scale [23], the Hoehn–Yahr scale, and disease duration. All patients were taking L-dopa or a DA agonist (carbidopa/levodopa equivalent daily dose). Patients with PD participated in behavioral experiments in the *On* condition, under which medicine was being administered.

**Table 1:** Participant details.

		HC	PD	*t*	*p*
Age (years)		68.65 (6.26)	71.90 (7.79)	1.453	0.155
Sex					
	Female	9	9		
	Male	11	11		
Hand dominance					
	Right	20	20		
	Left	0	0		
MMSE		27.35 (1.09)	27.00 (1.34)	0.101	0.920
MoCA		27.35 (1.23)	26.99 (1.08)	0.321	0.750
UPDRS		-	39.74 (27.65)		
LEDD			337.50 (167.71)		
Hoehn-Yahr stage		-	2.70 (0.86)		
PD duration (years)		-	7.25 (4.61)		

HC: elderly Healthy Control. PD: patient with Parkinson’s Disease. MMSE: Mini-Mental State Examination (max: 30). MoCA: Montreal Cognitive Assessment (max: 30). UPDRS: Unified Parkinson's Disease Rating Scale. LEDD: Levodopa Equivalent Daily Dose. The standard deviations are shown in parentheses.

### Behavioral measurements

We conducted a delayed reproduction task and estimation training task for time duration and line length. We used time durations of 11 s and 22 s, because these allowed us to investigate conscious behavior, unlike millisecond or circadian scales. Furthermore, previous researches clearly reported differences between healthy controls and PD patients using this time scale [18, 19]. The delayed reproduction task was conducted at the start. Three to four weeks later, we conducted an estimation training task. In both tasks, the participants were asked to estimate a specified time duration or line length with their right hand by holding a stylus pen above an electronic tablet (Intuos4 Extra Large, WACOM Corporation, Saitama, Japan; spatial precision, ±0.25 mm; sampling rate, 200 points/s, screen size, 488 mm × 305 mm; frame size, 623 mm × 462 mm). At the beginning of each trial, the duration or length to be estimated was verbally mentioned by the experimenter (S1 Video).

#### Delayed reproduction

**Fig 1:**
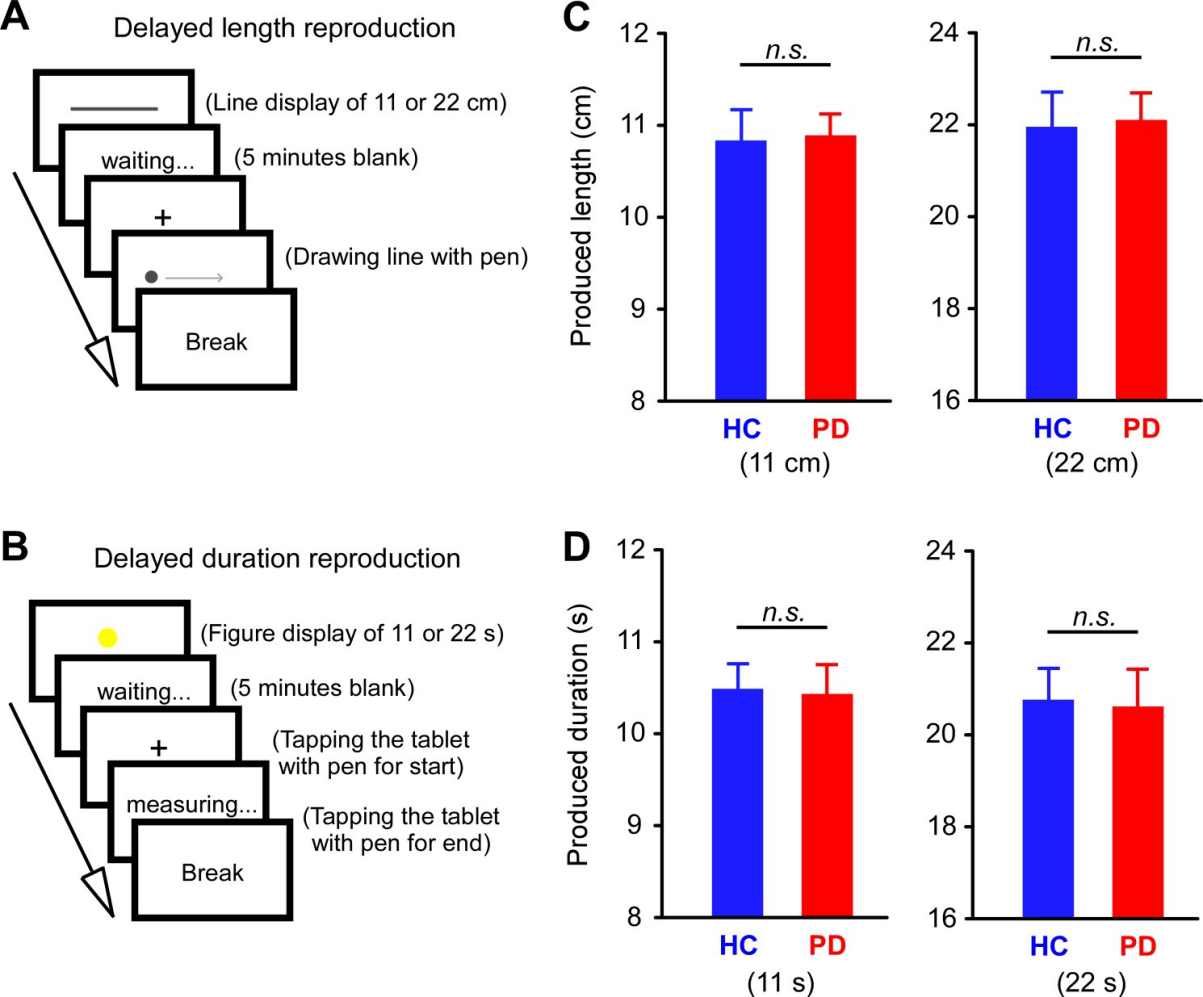
Delayed reproduction task. (**A**) Line length was estimated by drawing a line with a pen to reproduce line length (11 or 22 cm) after 5 minutes. The sample line was presented for 11 s. (**B**) Time duration was estimated by tapping a tablet with a pen to reproduce a sample presentation (11 or 22 s) after 5 minutes. (**C**) The estimated lengths did not differ between healthy controls (HC) and patients with Parkinson’s disease (PD) in the tasks involving both the 11 cm and 22 cm lines. (**D**) The estimated durations did not differ between the HC and PD groups in the tasks involving both 11 s and 22 s durations.

In the delayed reproduction task of length, the participants were asked to memorize and, after 5 minutes, to reproduce the length of a line on a computer screen (Fig 1A). The line was either 11 or 22 cm and was presented for 11 s. In the delayed reproduction task of duration, the participants were asked to memorize and, after 5 minutes, to reproduce the duration of presentation of a circle (Fig 1B). The circle was presented for either 11 or 22 s. The participants started each trial when they were ready and tapped the tablet with the pen at the beginning and end of the pen’s trajectory. Each task consisted of two trials, and intervals of minute-long were interposed between trials. The order was randomized between duration and length tasks.

#### Estimation training

**Fig 2:**
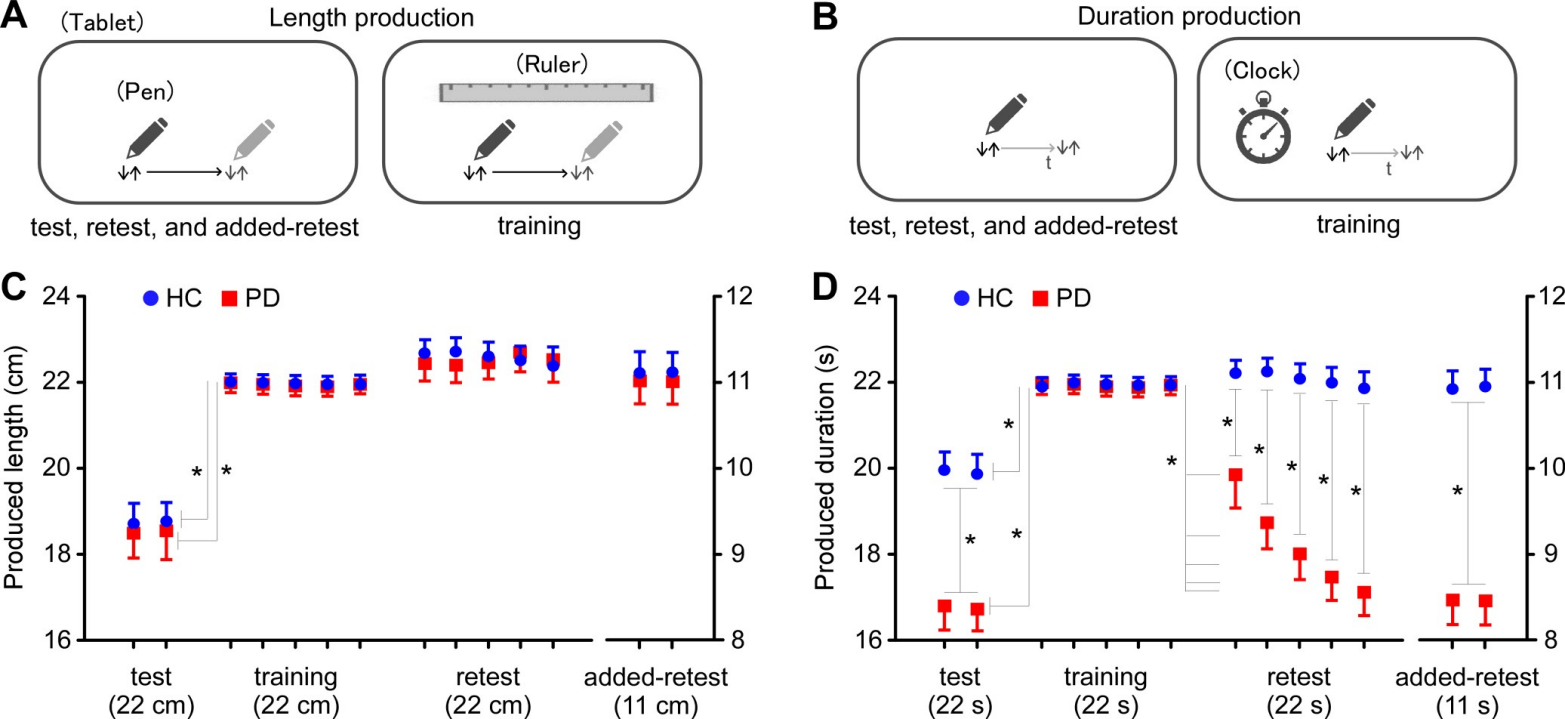
Estimation training task. (**A**) Line length was estimated by drawing a line with a pen without a cue in the test, retest, and added-retest sessions (left), and with a ruler cue in the training session (right). (**B**) Time duration was estimated by tapping the tablet with a pen without a cue in the test, retest, and added-retest sessions (left), and with a clock cue in the training session (right). (**C**) The estimated lengths did not differ between healthy controls (HC) and patients with Parkinson’s disease (PD) in any session. However, there were differences in performance between the second test and first training session in both HCs and patients with PD. (**D**) Estimated durations differed between the HC and PD groups in the test, retest, and added-retest sessions. Performance differed between the average of the test session and the first trial of the training session in both the HC and PD groups. Furthermore, the last trial of the training session differed from all trials in the retest session in the PD group.

In the length production task, the participants were asked to move the pen to the right for a length of 22 cm (Fig 2A). In the duration production task, the participants were asked to tap the tablet with a pen to start, wait for 22 s, and to tap the tablet again for the end of measurement (Fig 2B). The experiment consisted of four consecutive sessions: (1) test (two trials), (2) training (five trials), (3) retest (five trials), and (4) added retest (two trials). Intervals of minute-long were interposed between trials.

During the test, retest, and added-retest sessions, the participants completed the task without any cue. In the training session, a ruler cue for the length task and a clock cue for the duration task were provided in each trial. The ruler cue was a drawing of a ruler with its scale in millimeters and a maximum value of 30 cm. Where movement of the pen was required, the participants were asked to move it as constantly as possible and to keep it 5–10 mm above the surface of the tablet to avoid undesirable occasional contact to the surface because of tremor symptoms that must be considered in patients with PD. The clock cue was a drawing of an analog clock with its dial scaled in seconds, and a maximum value of 60 s. The second hand moved in smooth rotation as with a real clock. Finally, we repeated the duration/length estimation tasks for 11 cm and 11 s, respectively. This confirmed any generalization effect of estimation. The order was randomized between the length and duration tasks.

### Statistical analysis

Repeated measures analysis of variance (ANOVA) and *t* tests were conducted for behavioral performance and screening scores. Duration and length data were analyzed separately in both delayed reproduction and estimation training tasks. In addition, the 11/22 cm and 11/22 s conditions were analyzed independently. All tests were two-tailed. The results are shown as the mean ± standard error of mean and effect size (eta squared, *η*^2^). Statistical significance was defined as *p* < 0.05.

## Results

### Delayed reproduction

No significant differences were observed in the reproduced time durations and line lengths between the PD and HC groups (Fig 1C and 1D). Comparison between groups confirmed the absence of significant differences in reproduced duration/length across all conditions (S1 Table). Thus, all participants could reproduce both durations and lengths with a 5-minute delay.

### Estimation training

#### Length estimation

In the test session, the estimated length did not differ between the groups (*t*_38_ = 0.133, *p* = 0.895). Furthermore, the average length in test sessions was shorter than that in the first trial of the training session in both PD (*t*_19_ = 14.554, *p* < 0.0001) and HC (*t*_19_ = 12.349, *p* < 0.0001) groups (Fig 2C). Thus, PD did not appear to affect estimated lengths, although the lengths estimated by both PD and HC groups were shorter than the accurate length.

Repeated measures ANOVA showed no main effects of group (*F*_1, 38_ = 0.133, *p* = 0.718, *η*^2^ = 0.003), retest session (*F*_4, 152_ = 1.427, *p* = 0.228, *η*^2^ = 0.036), or the interaction (*F*_4, 152_ = 0.486, *p* = 0.746, *η*^2^ = 0.013). In addition, the length estimated in the last trial of the training session did not differ from that in all trials of the retest session in either PD or HC group (S2 Table). In contrast, the estimated lengths in all trials of the retest session were longer than those in the test session in both the PD (trial 1: *t*_19_ = 10.266 *p* < 0.0001; trial 2: *t*_19_ = 12.340, *p* < 0.0001; trial 3: *t*_19_ = 13.223, *p* < 0.0001; trial 4: *t*_19_ = 12.134, *p* < 0.0001; and trial 5: *t*_19_ = 12.168, *p* < 0.0001) and HC groups (trial 1: *t*_19_ = 9.437, *p* < 0.0001; trial 2: *t*_19_ = 10.987, *p* < 0.0001; trial 3: *t*_19_ = 11.934, *p* < 0.0001; trial 4: *t*_19_ = 13.139, *p* < 0.0001; and trial 5: *t*_19_ = 13.034, *p* < 0.0001). This suggests a training effect in both groups, and the effect was retained during the retest session.

In the added-retest session with estimation of an 11 cm line, the average estimated length in the PD group did not differ from that in the HC group (*t*_38_ = 1.678, *p* = 0.112). Data from twice in the added-retest session did not differ from the last trial of the retest session in either PD (*t*_19_ = 1.978, *p* = 0.089) or HC (*t*_19_ = 1.929, *p* = 0.081) group. This suggests that the estimated length was generalized, and the generalization effect was retained in the added-retest session.

#### Duration estimation

During time duration estimation, in the test session, patients with PD estimated a shorter duration than did HC (Fig 2D). Comparison between groups showed that the duration was significantly shorter in the PD group than in the HC group (*t*_38_ = 5.567, *p* < 0.0001). Furthermore, the comparison revealed that the average duration of the test session was shorter than that of the first trial in the training session in both PD (*t*_18_ = 11.006, *p* < 0.0001) and HC (*t*_19_ = 5.789, *p* < 0.001) groups. Thus, the estimated-duration in the HC group was shorter than the accurate duration estimated with the clock cue, while the estimated duration in the PD group was much shorter than the duration estimated with the clock cue.

In the PD group, the durations estimated in the five trials of the retest session were shorter than those in the last trial of the training session (trial 1: *t*_19_ = 2.965, *p* = 0.009; trial 2: *t*_19_ = 6.780, *p* < 0.0001; trial 3: *t*_19_ = 8.978, *p* < 0.0001; trial 4: *t*_19_ = 10.897, *p* < 0.0001; and trial 5: *t*_19_ = 14.56, *p* < 0.0001). In contrast, there was no difference in the HC group (all *p* > 0.05). In the retest session, the PD group estimated shorter durations than did the HC group.

Repeated measures ANOVA revealed main effects of group (*F*_1, 38_ = 86.522, *p* < 0.0001, *η*^2^ = 0.695), training effect on retest session (*F*_4, 152_ = 10.143, *p* < 0.0001, *η*^2^ = 0.221), and the interaction (*F*_4, 152_ = 7.913, *p* < 0.0001, *η*^2^ = 0.172). Post hoc tests showed that durations were shorter in the PD group than in the HC group for all trials in the retest session (trial 1, adjusted *p* < 0.001; trial 2, *p* < 0.0001; trial 3, *p* < 0.0001; trial 4, *p* < 0.0001; and trial 5, *p* < 0.0001). Furthermore, the durations in the retest session of the PD group were negatively correlated with the trial numbers (*r* = −0.991, *p* < 0.0001), whereas those in the HC group were not correlated with the trial number (*r* = −0.290, *p* = 0.635). In brief, the modified duration estimated by patients with PD returned gradually to the pre-training duration within a few minutes, while the HCs retained the duration during the retest session.

In the added-retest session of 11 s duration estimation, comparison between groups revealed that the average estimated duration was shorter in the PD group than in the HC group (*t*_38_ = 8.136, *p* < 0.0001). Data from twice in the added-retest session did not differ from the last trial of the retest session of patients with the PD (*t*_19_ = 1.778, *p* = 0.157) or HC group (*t*_19_ = 1.989, *p* = 0.068). This suggests that the estimated duration was generalized, and the generalization effect was retained in the added-retest session.

## Discussion

In the present study, both HCs and patients with PD had normal memory ability for both time duration and line length in the delayed reproduction task. Furthermore, HCs could modify their estimated duration/length during estimation training and retain these modifications for 5 minutes after training. However, patients with PD failed to learn the modified durations, but could retain the modified length estimation. Our findings suggest that PD impairs learning of duration specifically, rather than learning function more generally.

The lengths estimated by the patients with PD did not differ significantly from those estimated by HCs in the test and retest sessions. However, the patients with PD underestimated the durations in their sessions. Previous study has shown that reduced DA levels lead to hyperactivity or rapid signal cycling in the globus pallidus and subthalamic nuclei [24, 25]. Relatedly, fast mental counting of time (time compression) may be associated with hyper-activity or fast signal cycles in a loop system, such as the striatum–pallidus–thalamus–cortex loop [26], and extreme time compression can be consolidated due to prolonged symptoms in patients with PD, making it difficult to modify duration estimation.

Performance on the delayed reproduction task was normal in patients with PD. This task is thought to depend on memory ability. For example, when a patient estimated a duration of objective 22 s as being subjective 16 s, we rated the answer as accurate if the patient retained and reproduced the subjective 16 s duration. The underestimated durations in the test session may reflect truly dysfunctional time processing that is independent of memory ability in the delayed reproduction task [18]. Although patients with PD may be able to maintain their own duration easily, they may find it difficult to modify their duration. Thus, PD may not affect memory function for durations, but it may strongly affect the estimation and learning of durations.

Notably, durations estimated by patients with PD shortened gradually over several few minutes, indicating that the training effects were retained for a short time. This suggests that there is a time window during which the process of duration training is lost, and therefore that an effect of duration training may be supported by working memory [27, 28]. Previous studies have reported that working memory in patients with PD is dysfunctional [29]. However, patients in the present study accurately estimated durations during the delayed reproduction task. Thus, patients with PD underestimated duration but had normal memory for duration. It follows that, particularly in the first trial, the estimated duration in the retest session may indicate that the tendency of the “clock system to return to its original state” and the property of the “memory system to retain information” act antagonistically.

The present study had several limitations. Firstly, the dimensions of time duration and line length are not directly comparable, and the difficulty of the task also differed between the two. That is, the comparison between duration and length estimations may depend on the difficulty of task. To ascertain whether the deficits observed are a property of duration estimation specifically, experiments involving a variety of tasks will be necessary. Secondly, we used two screenings for mild cognitive impairment and suspicion of dementia, and the cutoff values were 26 points in both cases. The MoCA is much more sensitive than the MMSE in the classification of cognitive impairment in PD [30], so we might have adopted standards for respective cutoff values [31]. However, because a few patients had a lower score in the MMSE than in the MoCA in our past study, we excluded all patients at the lower limit of either test. This increased the probability that all participants with mild cognitive impairment or dementia were excluded. Finally, we did not assess the severity of depression in the present study. It has been reported that depression affects time estimation [32], and that patients with PD have a high incidence of depression [33]. Therefore, the influence of depression remains a potential confounder for the effect of PD on the learning of duration estimation.

The current study showed cognitive modification after training in duration estimation in HCs, suggesting that healthy people have the flexibility to alter awareness of duration. Future research should investigate how long these training effects can be maintained and whether these people can consolidate modified duration estimation in daily life. In contrast, the flexibility of duration estimation was lost in patients with PD, perhaps because of extreme time compression. Previous studies in patients with PD have reported that DA agonists lead to a shift in duration estimation towards normal [9], indicating that duration estimation is related to dopaminergic neurons. The current study suggested that PD impairs modification of duration estimation, while modification of length estimation remained possible. When impaired dopaminergic neurons affect cognitive function, learning disability may occur in the corresponding function. In this regard, it is known that PD impairs a recognition of facial expression [34]. Future research should address whether patients with PD show a learning impairment for recognition of facial expression. Finally, distorted duration estimation is reported in a wide variety of conditions, such as depression [32], epilepsy [35], schizophrenia [36], and attention-deficit hyperactivity disorder [37]. These conditions involve dopaminergic neurons, and the extension and reduction of duration estimation correspond to deficiency and excess of DA, respectively. Further investigation is required to elucidate the effect of these diseases on learning of duration estimation.

## Supporting information

S1 Video A representative trial of length estimation training without cues in a patient with Parkinson’s disease. (MP4)

S1 Table Group comparisons in the delayed reproduction task. (PDF)

S2 Table Differences in the length estimation training task between the last trial in the training session and the trials in the retest session. (PDF)
