# Supplementary material for: Impaired cognitive modification for estimating time duration in Parkinson’s disease
Source: PLoS One. 2018 Dec 13;13(12):e0208956. doi: 10.1371/journal.pone.0208956 (PMC6292599; doi:10.1371/journal.pone.0208956)
Supplement: S1 Table — (PDF) [file pone.0208956.s002.pdf]

Supporting Information

Impaired cognitive modification for estimating time duration in Parkinson's disease

Motoyasu Honma, Yuri Masaoka, Shinichi Koyama, Takeshi Kuroda, Akinori Futamura, Azusa Shiromaru, Yasuo Terao, Kenjiro Ono, and Mitsuru Kawamura

S1 Table. Group comparisons on delayed reproduction task.

Dimension	Condition	*df*	*t*	*p*
Distance	11 cm	38	0.600	0.552
	22 cm	38	0.135	0.893
Time-Duration	11 s	38	0.187	0.853
	22 s	38	0.541	0.592
