# Supplementary material for: Impaired cognitive modification for estimating time duration in Parkinson’s disease
Source: PLoS One. 2018 Dec 13;13(12):e0208956. doi: 10.1371/journal.pone.0208956 (PMC6292599; doi:10.1371/journal.pone.0208956)
Supplement: S2 Table — (PDF) [file pone.0208956.s003.pdf]

Supporting Information

Impaired cognitive modification for estimating time duration in Parkinson's disease

Motoyasu Honma, Yuri Masaoka, Shinichi Koyama, Takeshi Kuroda, Akinori Futamura, Azusa Shiromaru, Yasuo Terao, Kenjiro Ono, and Mitsuru Kawamura

S2 Table. Difference in performance between the last trial in training session with trials in retest session for estimation training task of distance.

Group	Trial	*df*	*t*	*p*
HC	First	19	0.246	0.808
	Second	19	1.954	0.066
	Third	19	1.683	0.109
	Forth	19	1.461	0.160
	Fifth	19	1.942	0.054
PD	First	19	0.813	0.427
	Second	19	0.888	0.386
	Third	19	1.925	0.070
	Forth	19	1.997	0.061
	Fifth	19	1.865	0.079
